# Influence of Parental Health Literacy on Change over Time in the Oral Health of American Indian Children

**DOI:** 10.3390/ijerph18115633

**Published:** 2021-05-25

**Authors:** Angela G. Brega, Rachel L. Johnson, Luohua Jiang, Anne R. Wilson, Sarah J. Schmiege, Judith Albino

**Affiliations:** 1 Centers for American Indian and Alaska Native Health, Colorado School of Public Health, University of Colorado Anschutz Medical Campus, Aurora, CO 80045, USA; judith.albino@cuanschutz.edu; 2 Department of Biostatistics and Informatics, Colorado School of Public Health, University of Colorado Anschutz Medical Campus, Aurora, CO 80045, USA; rachel.johnson@cuanschutz.edu (R.L.J.); sarah.schmiege@cuanschutz.edu (S.J.S.); 3 Department of Epidemiology & Biostatistics, Susan and Henry Samueli College of Health Sciences, University of California Irvine, Irvine, CA 92697, USA; lhjiang@hs.uci.edu; 4 Department of Pediatric Dentistry, School of Dental Medicine, University of Colorado Anschutz Medical Campus, Aurora, CO 80045, USA; anne.wilson@childrenscolorado.org

**Keywords:** health literacy, pediatric oral health, American Indian, longitudinal studies

## Abstract

In cross-sectional studies, parental health literacy (HL) is associated with children’s oral health. It is unclear, however, whether HL *influences* pediatric outcomes. We examined the relationship of HL with change over time in parental oral health knowledge, beliefs, and behaviors, as well as pediatric oral health outcomes. We used longitudinal data from a study designed to reduce dental decay in American Indian children (*N* = 579). At baseline and annually for three years, parents answered questions assessing HL; oral health knowledge, beliefs, and behaviors; and pediatric oral health status. The number of decayed, missing, and filled tooth surfaces (dmfs) was computed based on annual dental evaluations. Linear mixed models showed that HL was significantly associated with all constructs, except dmfs, at their reference time points and persistently across the three-year study period. HL predicted *change over time* in only one variable, parents’ belief that children’s oral health is determined by chance or luck. HL is strongly associated with oral health knowledge, beliefs, behaviors, and status prospectively but is not a key driver of change over time in these oral health constructs.

## 1. Introduction

Health literacy (HL) is “the capacity to obtain, process, and understand basic health information and services needed to make appropriate health decisions” [1]. The literature suggests that parental HL is linked to pediatric oral health. Compared to parents with stronger HL, parents with more limited HL have more restricted knowledge of children’s oral health and of oral health practices that are recommended for parents of young children [2,3,4,5]. Likewise, there is evidence that parents with more limited HL are less likely to hold oral health beliefs that are conducive to optimal oral health behaviors [3,5]. Specifically, compared to higher-literate parents, those with more limited HL perceive pediatric oral health problems to be less severe, perceive more barriers to and fewer benefits of recommended parental oral health behaviors, feel less confident that they can successfully engage in these practices, and are more likely to believe that their children’s oral health is under the control of the dentist or is simply a matter of chance or luck [3,5] and less likely to believe that they themselves are in control of their children’s oral health [3]. Perhaps as a result, parents with limited HL are less likely to follow recommended parental oral health practices (e.g., brushing child’s teeth with fluoride toothpaste) [3] and more likely to engage in behaviors that are harmful to pediatric oral health (i.e., nighttime bottle feeding, no daily brushing of child’s teeth) [2]. Compared to parents with higher levels of HL, lower-literate parents have children with worse oral health outcomes, including poor oral health status [2,6], poor oral health-related quality of life [3], and dental decay [7,8].

Although the literature demonstrates a relationship between parental HL and pediatric oral health, prior studies have relied almost exclusively on cross-sectional designs. Hence, it is unclear whether parental HL actually *influences* children’s oral health over time. Theoretical models developed to clarify the relationship between HL and outcomes suggest that limited HL plays a causal role, impairing outcomes by making it difficult for individuals to develop appropriate knowledge, health beliefs, and behaviors [9]. The objective of this analysis was to test these expectations in the context of pediatric oral health. We hypothesized that limited parental HL serves as a barrier to the development of strong oral health knowledge, positive oral health beliefs, and optimal oral health behaviors in parents, and to the achievement of good oral health outcomes in children. 

We tested these hypotheses using data from a randomized controlled trial designed to reduce dental decay in American Indian (AI) children. Indigenous children have the highest rate of dental decay of any pediatric population in the United States [10]. Indeed, 71% of three- to five-year-old Indigenous children have dental decay, compared to 25% of non-Hispanic white children of the same age [10]. Dental decay is also more severe in Indigenous children. On average, Indigenous preschoolers aged two to five have four teeth that show signs of decay or that have been filled by a dentist, compared to an average of one tooth that is decayed or filled among non-Hispanic white children [11]. 

As a result of treaties, legislation, and legal decisions, the United States government is responsible for providing health care services, including dental care, to members of federally recognized Indigenous tribes. Yet, chronic funding and staffing shortages result in many AI communities having limited access to dental care [12,13]. As a result, Indigenous children have high rates of untreated dental decay. Compared with 10% of three- to five-year-old non-Hispanic white children in the United States, 43% of Indigenous children in the same age group have dental decay that is untreated [10].

Given high rates of dental decay and limited access to dental care, effective parental management of children’s oral health is particularly important in Indigenous communities. Yet, limited HL—estimated to affect 48% of Indigenous adults in the United States [14]—may make it difficult for AI parents to successfully manage their children’s oral health. Although parental HL may play an important role in pediatric oral health in all populations, the vast disparities in oral health and limited access to care experienced by Indigenous children makes the assessment of this connection particularly important in the Indigenous population of the United States. 

In this secondary analysis, we examined the association of parental HL with change in parental oral health knowledge, beliefs, and behaviors, as well as pediatric oral health outcomes over a three-year period in Indigenous families. As the first known study to explore the association of HL with *change over time* in these constructs, the reported analysis contributes important insight into the role that parental HL plays in pediatric oral health. Likewise, through its examination of oral health outcomes in Indigenous children—a group that is at high risk for poor outcomes and has limited access to dental care—this work fills an important gap in our understanding of pediatric oral health in AI populations.

## 2. Materials and Methods

### 2.1. Source of Secondary Data

In this secondary analysis project, we used data from the randomized controlled trial entitled “Promoting Behavioral Change for Oral Health in American Indian Mothers and Children” (PBC) [12,15]. The PBC study tested a behavioral intervention aimed at reducing dental decay in AI children from birth to three years of age. Early intervention to prevent oral health problems is recommended by the American Academy of Pediatric Dentistry [16,17] and is particularly important for Indigenous children, who develop teeth at an earlier age than non-Indigenous children [18] and have a high prevalence of dental decay in the early years of life (e.g., 61% of 3 year olds) [10].

### 2.2. PBC Participants and Procedures

As part of the PBC study, 579 parent–child dyads were enrolled, randomized, and followed for three years. At enrollment, participating children were required to be AI, 0–3 months of age, residing on or near the participating AI reservation, and free of medical conditions that could negatively affect tooth development. Adults were required to be the mothers or primary caregivers of participating children, 15–65 years of age, willing and able to follow the study protocol, and able to provide informed consent. 

Parent–child dyads were randomly assigned to the intervention group or the control group [12,15]. Parents in the intervention group participated in four motivational interviewing sessions aimed at identification and resolution of barriers to recommended parental oral health behaviors [19,20]. The control group received “enhanced community services”, which involved exposure to educational information disseminated across the reservation (e.g., public service announcements; educational brochures). The intervention resulted in improved parental oral health knowledge at the 12- and 24-month time points, but not at 36 months. It did not result in improved parental oral health behavior or pediatric oral health outcomes [12].

### 2.3. Institutional Review

This secondary analysis and the original PBC study were approved by the research review board of the participating tribe and the Colorado Multiple Institutional Review Board at the University of Colorado Anschutz Medical Campus. Parents provided written informed consent and Health Insurance Portability and Accountability Act (HIPAA) authorization prior to participation. For parents under 18 years of age, consent and authorization were provided by their own parents or legal guardians. 

### 2.4. Measures

At baseline and when children were 12-, 24-, and 36-months old, parents completed the Basic Research Factors Questionnaire (BRFQ) [21]. The BRFQ assessed parents’ HL; parental oral health knowledge, beliefs, and behavior; parents’ assessment of children’s oral health status; and sociodemographic characteristics. The items used in this analysis have been validated in Indigenous populations [22,23,24,25].

Dental evaluations were conducted when children were 12-, 24-, and 36-months old. Given that teeth begin to erupt ≈ 6 months of age, dental evaluations were not conducted at baseline, when children were newborns. Evaluations used standardized scoring criteria [26,27] and were conducted by licensed dental providers, who had been trained and calibrated [28] and were blind to treatment group.

#### 2.4.1. Health Literacy

Baseline HL was measured using three BRFQ items assessing parents’ confidence in their ability to read and complete health-related forms. Items were adapted from questions known to accurately identify patients with inadequate HL [29,30,31,32,33,34,35]. The overall HL score was the mean of the three items and had a range of 1–5, with larger numbers indicating stronger HL. 

#### 2.4.2. Parental Oral Health Knowledge

Oral health knowledge was measured using 17 BRFQ items assessing parents’ understanding of pediatric oral health (e.g., “Because they do not stay in your child’s mouth very long, baby teeth are not that important”) and recommended parental oral health practices (e.g., “It is best to use toothpaste with fluoride when brushing a child’s teeth”). Responses were coded as correct or incorrect. The overall knowledge score was the percentage of questions answered correctly. 

#### 2.4.3. Parental Oral Health Beliefs

The BRFQ contained items assessing health beliefs theorized to be important predictors of health behavior, including five constructs from the extended Health Belief Model (HBM) and three constructs addressing locus of control (LOC).

**Extended Health Belief Model.** According to the extended HBM [36,37], parents are more likely to engage in recommended parental oral health behaviors if they perceive their children to be susceptible to dental decay, perceive dental decay to be a severe outcome, perceive there to be few barriers and many benefits to recommended behaviors, and perceive themselves to be capable of engaging in these behaviors (i.e., self-efficacy). 

The BRFQ included three to five items each assessing perceived susceptibility, severity, barriers, and benefits. Although three susceptibility items were included in the BRFQ, one was excluded from the susceptibility score as it was not well correlated with the others. The average score for items associated with each construct was computed. Scores ranged from 1 to 5, with larger numbers reflecting greater endorsement of beliefs associated with the construct.

The BRFQ included 14 items assessing self-efficacy. Each item measured parents’ confidence that they could engage in a specific oral health behavior recommended for parents of young children (e.g., “How sure are you that you can take your child to the dentist for regular check-ups?”). For most items, a majority of parents selected the highest possible score (5 on the 1 to 5 scale). The mean of the items was, therefore, highly skewed (skew across all time points = −1.65). Hence, rather than computing the mean score, we calculated the number of questions for which the highest score was selected, consistent with our prior research [5]. This overall self-efficacy score was less skewed than the mean (skew = −0.69) and had a possible range of 0–14, with larger numbers reflecting greater self-efficacy. 

**Locus of Control (LOC).** According to Social Learning Theory, parents who feel a sense of control over their children’s oral health are more likely to engage in behaviors that lead to positive outcomes [38,39]. Nine BRFQ items, which were adapted from existing measures [40,41], were used to compute three measures of LOC. Respectively, these measures assessed parents’ belief that they themselves were in control of their children’s oral health (internal LOC), that the dentist was in control (external LOC—powerful others), and that their children’s oral health was a matter of chance or luck (external LOC—chance). For each type of LOC, the average of the three relevant items was computed. Overall scores ranged from 1 to 5, with larger numbers indicating stronger endorsement of items related to the specific type of LOC.

#### 2.4.4. Adherence to Recommended Parental Oral Health Behaviors

The BRFQ contained 13 items measuring adherence to oral health behaviors recommended for parents of young children (e.g., “When your child’s teeth are brushed, is fluoride toothpaste usually used?”) [16,17]. Responses were coded as adherent or non-adherent. The overall behavior score was the percentage of behaviors for which a parent was adherent.

#### 2.4.5. Oral Health Outcomes

We examined two measures of pediatric oral health. First, the BRFQ included a single item adapted from the National Survey of Children’s Health [42] that asked parents to rate their children’s oral health status as excellent (1), very good (2), good (3), fair (4), or poor (5). Second, from dental evaluation data, we computed the number of decayed, missing, and filled tooth surfaces (dmfs), a common and objective measure of a child’s oral health.

#### 2.4.6. Sociodemographic Characteristics

The BRFQ also assessed sociodemographic characteristics. For parents, the survey collected age, gender, race, tribe, ethnicity, highest grade completed, household income for the prior year, and employment status. For children, items assessed age, gender, race, and ethnicity.

### 2.5. Statistical Methods

Analyses were conducted in two steps. First, because our goal was to determine whether HL was associated with change over time in parental oral health knowledge, beliefs, and behaviors as well as pediatric oral health outcomes, we began by determining whether there were, in fact, significant changes over the three-year study period in these oral health constructs. Second, we examined whether parental HL was a significant predictor of these changes. As described below, analyses controlled for important covariates and addressed the potential for intervention group differences.

#### 2.5.1. Change over Time in the Oral Health Constructs

To determine whether there was change over time in the oral health constructs, linear mixed models were fit to all continuous measures with an unstructured covariance matrix and time as a categorical predictor. We also tested other potential covariance structures that could account for the intraclass correlations among repeated measures of the oral health constructs, including compound symmetry and first order autoregressive covariance structures. None of these alternate covariance structures fit the data better than the unstructured covariance, as determined by the Akaike information criterion. Hence, we retained the unstructured covariance matrix in the final models.

Since dmfs was a highly skewed variable, having a high proportion of zeros, a generalized linear mixed model was used to fit dmfs with a negative binomial distribution and a random intercept. We repeated these models using a first order autoregressive or unstructured covariance matrix and a Poisson distribution. None of these alternate approaches fit the data better than the initial model, which was retained as the final model. 

#### 2.5.2. Association of Health Literacy with Change over Time in the Oral Health Constructs

To determine whether HL was a significant predictor of change in the oral health constructs, we conducted the longitudinal regression models described above, adding baseline HL and the interaction between HL and time to the models. To allow for clearer interpretation of model estimates, z-scores were calculated to standardize the HL variable.

#### 2.5.3. Data Analysis Time Points

For all but two of the oral health constructs, we used data from four time points (i.e., baseline and when children were 12-, 24-, and 36-months old). For these constructs, the baseline time point served as the reference value in analyses. For the behavioral adherence and dmfs measures, we used data from three time points (i.e., 12, 24, and 36 months). Because the behavior items collected at baseline differed from those collected at the follow-up time points, we excluded baseline behavioral data and used 12-month data as the reference value in analyses. Given that children were enrolled as newborns, dental evaluations were not conducted at baseline. Hence, dmfs data were available only at the three follow-up time points and data from the 12-month visit served as the reference value in analyses.

#### 2.5.4. Covariates

All regression models controlled for parents’ age and income at baseline and for children’s gender. Age and income are associated with HL and, therefore, are commonly included as covariates in HL research [14]. Gender is associated with pediatric oral health, with boys typically experiencing worse dental disease than girls [43,44]. Therefore, child gender was included as a covariate in regression analyses. 

To determine whether analyses should control for treatment group, we examined whether the changes over time in the oral health constructs differed for participants in the clinical trial’s two treatment arms. To make this determination, we estimated the regression models that examined the presence of change over time in the oral health constructs again (see Section 2.5.1), adding treatment group (control or intervention) and the interaction between treatment group and time to each model. As shown in Supplemental Table S1, models showed significant differences in the pattern of change over time between treatment groups for two variables: parental oral health knowledge (*p* = 0.022) and external LOC—powerful others (*p* = 0.043). Hence, in the final models for these two constructs, we included treatment group and the interaction between treatment group and time as covariates.

## 3. Results

### 3.1. Sample Characteristics

Demographic characteristics for the sample are summarized briefly, as they have been described previously [5]. At baseline, parents were 25.0 years old (SD 5.5), on average. Nearly all were female (97.2%) and most were mothers of enrolled children (95.9%). The vast majority of parents were AI (95.2%), with three-quarters (73.7%) identifying as members of the Northern Plains tribe that participated in the clinical trial. Among parents, 40.1% had less than a high school education, 51.1% reported a household income less than $10,000 for the prior year, and unemployment was common (50.3%). At baseline, the average age of enrolled children was 0.7 months (SD = 0.9), or ≈3 weeks old. Children were evenly split by gender (51.1% female) and all were AI.

### 3.2. Initial Performance on Constructs of Interest

Table 1 presents initial performance on HL and each oral health construct at the appropriate reference time point. At baseline, parents were relatively confident in their ability to read and complete medical forms (mean = 3.9 on a scale of 1 to 5). Likewise, parental oral health knowledge was relatively strong, with parents answering 75.7% of questions correctly, on average. On a scale of 1 to 5, parents did not perceive their children to be highly susceptible to dental problems (mean = 2.9). However, they did believe that dental decay was a fairly severe outcome (mean = 4.4) and that there were many benefits (mean = 4.4) and few barriers (mean = 2.1) to recommended parental oral health practices. 

At baseline, parents felt a good deal of control over their children’s oral health. Most felt confident that they could engage in recommended parental oral health behaviors (mean = 9.1 out of 14 behaviors). On a scale of 1 to 5, parents typically reported that they had control over their children’s oral health (mean = 4.1) and did not strongly endorse the belief that their children’s outcomes were up to the dentist (mean = 2.2) or to chance (mean = 2.4). At the 12-month reference time point, parents engaged in slightly less than half of recommended parental oral health behaviors (mean = 49.2%). 

Early in the PBC study, oral health outcomes were quite positive. At baseline, parents reported the oral health status of their children to be good (mean = 1.6 on a scale of 1–5, with lower numbers reflecting better oral health). At the 12-month reference time point, mean dmfs was 0.4, reflecting a small number of tooth surfaces affected by decay.

### 3.3. Change over Time in the Oral Health Constructs

Several oral health constructs showed significant improvement over time (Table 1). Oral health knowledge was significantly stronger at each follow-up time point, compared to baseline (∆ ranged from 3.12 to 4.74, *p* < 0.001 for all changes). Over time, parents also developed more positive attitudes regarding the source of control over their children’s oral health. Parents showed a significant increase in internal LOC from baseline to 12 months (∆ = 0.12, *p* = 0.010), a significant decline in external LOC—powerful others at each time point (∆ ranged from −0.24 to −0.35, *p* < 0.001 for all changes), and a significant decline in external LOC—chance from baseline to 24 months (∆ = −0.11, *p* = 0.026). 

Results also showed deleterious changes over time (Table 1). From baseline to 36 months, parents perceived significantly greater barriers to recommended parental oral health behaviors (∆ = 0.09, *p* = 0.038). Compared to the reference time point, significant worsening also was seen at each subsequent time point in behavioral adherence (∆ ranged from −7.71 to −7.85 points, *p* < 0.001 for all changes). Likewise, oral health status worsened over time (∆ ranged from 0.31 to 1.14, *p* < 0.001 for all changes), as did dmfs (∆ ranged from 15.63 to 68.92, *p* < 0.001 for all changes). 

### 3.4. Association of Health Literacy with Change over Time in the Oral Health Constructs

To determine whether baseline HL affected change over time in the oral health constructs, baseline HL (standardized), time, and the interaction between HL and time were included in the regression models. The complete results of this analysis are reported in Supplemental Table S2; Table 2 highlights the results related to HL and the HL by time interaction. As shown in Table 2, baseline HL was strongly associated with all but one of the oral health constructs at the reference time point (i.e., 12 months for behavior and dmfs; baseline for all other variables). Specifically, it was significantly associated with parental oral health knowledge, beliefs, behaviors, and pediatric oral health status (*ps* ranged from 0.004 to < 0.001) but was not associated with dmfs (*p* > 0.05).

As shown in Table 2, the interaction between HL and time was statistically significant for a single oral health construct: external LOC—chance (*p* < 0.001). Examining the main effects that contributed to this interaction, we found that—at baseline—parents with more limited HL were significantly more likely than higher-literate parents to endorse the belief that their children’s oral health was a matter of chance (*β* = −0.29, *p* < 0.001). As shown in Supplemental Table S2, there was not a significant main effect of time (*p* > 0.05). That is, for those with average HL at baseline, we did not see significant change over time in endorsement of this belief. However, there was a significant interaction between HL and time, with external LOC—chance scores changing significantly more from baseline to 24 months (*β* = 0.22, *p* < 0.001) and baseline to 36 months (*β* = 0.14, *p* = 0.009) among parents with more limited HL at baseline (see Supplemental Table S2).

**Figure 1:**
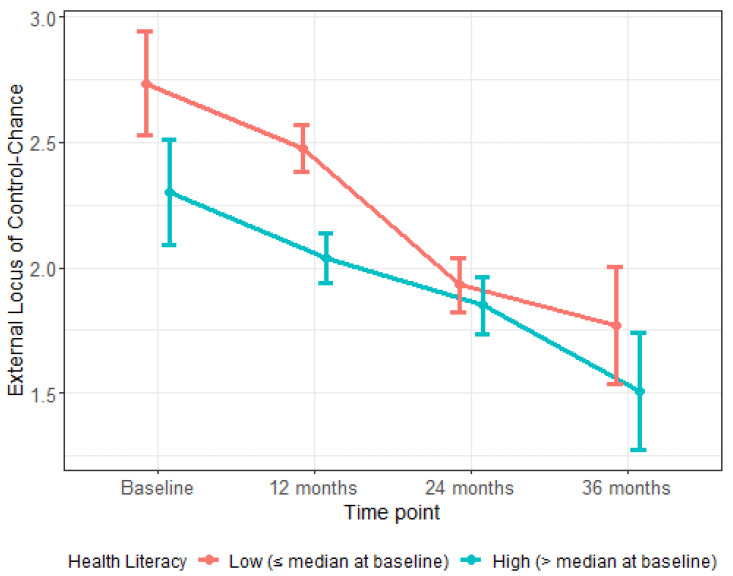
Health Literacy and Change over Time in External Locus of Control—Chance. The figure displays the association of baseline health literacy with change over time in external locus of control—chance, adjusted for parent’s age and income as well as child’s gender. The two lines depict endorsement of the belief that children’s oral health is a matter of chance in two group: parents with health literacy levels that are at or below the median for the sample at baseline and parents whose health literacy levels are above the median for the sample at baseline.

Figure 1 provides additional insight into this interaction. The figure depicts endorsement over time of the belief that children’s oral health is a matter of chance for two groups of parents: those with baseline HL scores at or below the median for the sample (“low HL”) and those with baseline HL scores above the median (“high HL”). Compared to parents with high HL, those with low HL more strongly endorsed the belief that children’s oral health was up to chance at all time points. Ad hoc regression analyses showed that parents with low HL were significantly more likely than parents with high HL to endorse this belief at baseline (*β* = −0.44, *p* < 0.0001), 12 months (*β* = −0.44, *p* < 0.0001), and 36 months (*β* = −0.26, *p* = 0.006). The groups did not differ at 24 months (*p* > 0.05). 

Table 3 presents the results of additional ad hoc regression analyses modeling external LOC—chance scores over time with an interaction between HL group (low vs. high) and time. Results showed that external LOC—chance scores did not change significantly over time among parents with high HL (*ps* > 0.05). For parents with low HL, however, scores declined significantly from baseline to 24 months (*β* = −0.26, *p* < 0.001) and baseline to 36 months (*β* = −0.15, *p* = 0.023). 

## 4. Discussion

The literature suggests that HL is associated with oral health-related knowledge, beliefs, and behavior among parents, and oral health outcomes in children [2,3,4,5,6,7,8,45,46,47,48,49]. These results—along with similar findings from studies examining HL in other health conditions [50,51,52,53]—have led many to propose that HL may play a causal role in health [9]. Yet, reliance on cross-sectional data has limited our ability to evaluate whether HL *influences* health or is simply correlated with health-related constructs at a single point in time.

The objective of this analysis was to determine whether limitations in parental HL play a role in the development of dental decay among Indigenous children. Over the three-year study period, participants experienced significant change in oral health knowledge, specific beliefs, behavior, and outcomes. Although baseline HL was strongly associated with all but one of the oral health constructs at their reference time points (i.e., dmfs), it was a significant predictor of *change over time* in only one variable (i.e., external LOC—chance). That the main effect of HL was significant in the longitudinal models for nearly all constructs indicates that parental HL was *persistently* associated with oral health knowledge, beliefs, behavior, and outcomes across the three-year study period. Importantly, because HL was measured at baseline while all other constructs were measured longitudinally starting at baseline or the 12-month follow-up visit, our findings also indicate that HL was *prospectively* associated with these constructs. Although this analysis cannot demonstrate causality, it provides strong evidence that HL predicts subsequent oral health knowledge, beliefs, behavior, and outcomes.

As noted, parental HL was associated with change over time for the external LOC—chance variable. Parents with low HL experienced a significant decline in the perception that their children’s oral health was a matter of chance, whereas parents with high HL showed no change. Given that parents with low HL endorsed this belief to a significantly greater degree at baseline than did parents with high HL, it is possible that the greater decline among parents with low HL occurred simply because they had more room for improvement. However, our finding of an association between HL and parents’ sense of control over pediatric oral health is consistent with prior research. In cross-sectional analyses of data from the PBC sample and a sample of Navajo families, parents with stronger HL reported a greater sense of control over their children’s oral health. They were more likely to believe that their children’s oral health was under their control and less likely to believe it was under the control of the dentist or was up to chance [3,5]. Likewise, Navajo parents with stronger HL felt more confident in their ability to engage in recommended parental oral health behaviors than did parents with more limited HL [3]. Similarly, in a path analysis designed to clarify the mechanisms underlying the relationship between HL and parental oral health behavior in the PBC sample, HL had a significant indirect effect on behavior, through self-efficacy [54]. Our finding of a significant main effect of HL on external LOC—chance is consistent with this earlier evidence that HL is linked to parents’ sense of control over children’s oral health.

Our results suggest, however, that limited HL does not preclude the development of a sense of control. Indeed, although parents with high HL showed no change in the belief that their children’s oral health was a matter of chance, parents with low HL showed a significant decline in this belief. Although this finding was not expected, it is consistent with prior research related to the role of HL in development of health-related knowledge. In a recent intervention study [55], investigators found that women with more limited HL had lower oral health knowledge scores both before and after intervention, compared to women with stronger HL. In an intervention arm designed to be sensitive to HL limitations, however, knowledge scores improved more among lower-literate than higher-literate women. Although this finding may be due to the fact that knowledge scores of lower-literate women had more room for improvement, it is striking that this pattern was seen only in a specific treatment arm. Similarly, in a study examining the association of HL with development of cardiovascular knowledge among Indigenous people [56], participants with more limited HL showed the same degree of improvement in knowledge over time as did higher-literate participants. Yet, knowledge scores were significantly worse among lower-literate participants at both the baseline and follow-up time points. In combination, these results suggest that limited HL may be associated with lower levels of knowledge and positive health beliefs but may not serve as an intractable barrier to the development of knowledge and optimal beliefs. These results do suggest, however, that lower-literate individuals may require higher-intensity intervention to achieve levels of knowledge and positive oral health beliefs experienced by individuals with stronger HL. 

The research we report has important implications for understanding the role of parental HL in the oral health outcomes of young Indigenous children. Our findings suggest that lower HL is associated with but does not impede parents’ acquisition of oral health knowledge or the development of positive oral health beliefs and behaviors. Nor does it appear to consign children of affected parents to poor oral health outcomes. Indeed, in combination with findings from prior studies [55,56], this work suggests that HL may play an important role with regard to parents’ sense of control over oral health behaviors and outcomes. 

To fully understand our results, however, it is important to consider the social context in which the PBC trial was conducted. Participating families experienced limited educational opportunities and extreme economic hardship. Forty percent of parents had less than a high school education, with more than half reporting being unemployed and having a household income of less than $10,000. These indicators of socioeconomic adversity are consistent with life on the participating reservation, where scarcity in educational and economic opportunities is widespread [57,58]. Importantly, the income, education, and employment challenges faced by participating families are known predictors of poor oral health outcomes in children [59]. 

Access to dental care is another key determinant of pediatric oral health [59]. Oral health guidelines highlight the importance of establishing a dental home and beginning routine dental visits before a child reaches 12 months of age [16]. Yet, access to dental services is limited on the reservation where the PBC trial was conducted. Although the reservation is home to ≈20,000 residents [58] and covers an area nearly the size of Connecticut, it has only three dental clinics. Likewise, difficulty filling dental positions results in a dentist-to-patient ratio much worse than seen nationally (1:4000 vs. 1:1600) [12,13]. As a result, access to dental care on the reservation is limited.

In the face of these challenges, perhaps it should not be a surprise that parental HL was not a significant predictor of change over time in most of the oral health constructs. Indeed, it is conceivable that strong HL may play a more substantial role in facilitating positive outcomes in contexts where parents are genuinely capable of engaging in optimal health behaviors. Material deprivation and lack of access to dental services may prevent parents from taking recommended actions, even when they fully understand how to care for their children’s teeth. In the PBC trial, systemic barriers to optimal parental oral health behavior may simply have outweighed the potential impact of parental HL. 

The work reported here had important strengths. First, the analysis approach allowed us to examine the temporal precedence of HL with regard to the oral health constructs. Although this technique cannot definitively demonstrate causation, it allowed us to assess the possibility that parental HL actually influences pediatric oral health. Second, this analysis was strongly grounded in accepted health behavior theory [36,37,39] as well as research and theoretical work in the HL field [9,50,51]. Finally, this work provided insight into the relationship of parental HL with pediatric oral health in a population that is at risk for poor oral health outcomes [10,11,13,60,61] and is rarely targeted in oral health research.

Like all studies, the reported analysis also had limitations. First, this work focused on a single health condition, pediatric oral health. It is possible that HL may function differently in the context of other medical conditions. Indeed, this could explain why there is not perfect agreement across studies as to the association of HL with health-related behaviors and outcomes [50,51]. Second, this work focused on a single population, AIs living on or near a specific reservation in the Northern Plains. Especially given the extreme economic hardship and limited access to dental care experienced by this population, our results may not generalize to other groups. Third, the measure of HL used in this study assessed parent’s subjective perceptions of their ability to read and write in the context of health. Although the items were adapted from well-tested screening questions [29,30,31,32,33,34,35] and have been validated in Indigenous populations [24], they do not capture the full complement of skills considered to be components of HL (e.g., numeracy skills, verbal communication skills). Finally, we examined the association of HL with change over time in several constructs. It is possible that our finding of a significant interaction between HL and external LOC—chance was an artifact of the large number of relationships examined.

## 5. Conclusions

### 5.1. Background and Summary of Findings

Conceptual models designed to clarify the connections between HL and health propose that limited HL serves as a barrier to the development of health-related knowledge, optimal health beliefs and behaviors, and positive health outcomes [9]. Yet, heavy reliance on cross-sectional data in the field has made it difficult to gauge the degree to which HL prospectively influences health [50]. In the longitudinal analysis reported here, we sought to address this limitation in the literature and to shed light on the direct contribution of parental HL to children’s oral health outcomes. The reported analysis indicated that baseline HL was associated with oral health knowledge, beliefs, behaviors, and status prospectively and persistently over the three-year study period. It did not, however, influence change over time in most of these constructs. For only one construct—the perception that one’s child’s oral health is a matter of chance—did parental HL affect change over time. In all, these findings suggest that limited HL is not a barrier to the development of strong oral health knowledge, optimal beliefs and behaviors, and good oral health status, but that it is strongly associated with these constructs both cross-sectionally and prospectively.

It is possible that the social context in which participating families lived may have overshadowed the potential impact of HL on change over time in the oral health constructs. Families enrolled in the PBC study experienced high rates of unemployment as well as limited educational attainment and income. These factors, in combination with the limited availability of dental care on the participating reservation, may have constrained the importance of HL as a potential driver of oral health outcomes. Even parents with strong HL, excellent knowledge, and positive oral health beliefs may not have been able to engage in recommended oral health behaviors in this context (e.g., obtaining routine dental check-ups, paying for toothbrushes and toothpaste). Similar research in communities less affected by material deprivation and limited access to dental care may show different results.

### 5.2. Future Research Directions

The reported analysis provides important insight into the role of parental HL in pediatric oral health. That said, important questions remain. Although this work suggests that HL is strongly associated with oral health knowledge, beliefs, behaviors, and status, it does not clarify the mechanisms underlying these relationships. A critical next step is to test a comprehensive conceptual framework designed to clarify the pathways through which parental HL is linked to these constructs. Is HL related to beliefs through its association with knowledge, for instance? Is it associated with oral health status as a result of its connection to behavior? Use of path analysis or structural equation modeling to examine the longitudinal pathways connecting these constructs would significantly advance the science of HL and inform the work of clinical providers and health intervention developers seeking to address the potential impact of HL on oral health.

## Figures and Tables

**Table 1:** Initial Performance and Change over Time in Constructs of Interest ^1^.

Construct	Mean (SD) ^2^	Adjusted Time Estimate (∆) (95% CI)	*p* Value
Health Literacy	3.9 (0.8)	---	---
Parental Oral Health Knowledge	75.7 (12.9)		<0.001
12 Months		3.12 (2.02, 4.22)	<0.001
24 Months		3.49 (2.19, 4.78)	<0.001
36 Months		4.74 (3.52, 5.97)	<0.001
Parental Oral Health Beliefs Extended Health Belief Model			
Perceived Susceptibility	2.9 (1.0)		0.271
12 Months		−0.09 (−0.19, 0.01)	0.079
24 Months		−0.04 (−0.14, 0.06)	0.399
36 Months		−0.01 (−0.11, 0.10)	0.913
Perceived Severity	4.4 (0.8)		0.078
12 Months		0.03 (−0.04, 0.10)	0.469
24 Months		0.00 (−0.07, 0.08)	0.913
36 Months		−0.07 (−0.15, 0.01)	0.082
Perceived Barriers	2.1 (0.9)		0.143
12 Months		0.04 (−0.04, 0.12)	0.364
24 Months		0.02 (−0.06, 0.10)	0.646
36 Months		0.09 (0.01, 0.17)	0.038
Perceived Benefits	4.4 (0.8)		0.484
12 Months		0.05 (−0.04, 0.13)	0.284
24 Months		0.04 (−0.04, 0.11)	0.357
36 Months		0.06 (−0.02, 0.15)	0.127
Self-efficacy	9.1 (4.1)		0.547
12 Months		0.13 (−0.20, 0.46)	0.431
24 Months		−0.10 (−0.48, 0.28)	0.598
36 Months		−0.05 (−0.43, 0.33)	0.795
Locus of Control (LOC)			
Internal LOC	4.1 (0.9)		0.012
12 Months		0.12 (0.03, 0.21)	0.010
24 Months		0.00 (−0.10, 0.10)	0.980
36 Months		0.05 (−0.05, 0.10)	0.338
External LOC—Powerful Others	2.2 (1.1)		<0.001
12 Months		−0.24 (−0.33, −0.14)	<0.001
24 Months		−0.35 (−0.45, −0.26)	<0.001
36 Months		−0.29 (−0.40, −0.19)	<0.001
External LOC–Chance	2.4 (1.1)		0.073
12 Months		0.00 (−0.09, 0.09)	0.970
24 Months		−0.11 (−0.21, −0.01)	0.026
36 Months		−0.07 (−0.17, 0.03)	0.151
Parental Oral Health Behavior ^3^	49.2 (23.3)		<0.001
24 Months		−7.71 (−9.56, −5.88)	<0.001
36 Months		−7.85 (−9.74, −5.96)	<0.001
Pediatric Oral Health Outcomes			
Oral Health Status	1.6 (0.9)		<0.001
12 Months		0.31 (0.21, 0.41)	<0.001
24 Months		0.92 (0.80, 1.05)	<0.001
36 Months		1.14 (1.00, 1.27)	<0.001
dmfs ^3^	0.4 (1.9)		<0.001
24 Months		15.63 (10.63, 22.98)	<0.001
36 Months		68.92 (46.49, 102.17)	<0.001

^1^ For each construct, the table displays the estimate comparing each time point to the reference time point. Also displayed are the 95% confidence interval (CI), *p* value for the comparison of each time point to the reference value, and the overall *p* value across all time points. Models were adjusted for parental age and income at baseline as well as child’s gender. ^2^ The table presents the mean and standard deviation (SD) for each construct at the appropriate reference time point. For all but two constructs—behavior and dmfs—the reference value was from the baseline time point. Because behavior items collected at baseline differed from those collected at the follow-up time points, we excluded baseline behavioral data and used 12-month data as the reference value. Given that children were enrolled as newborns, dental evaluations were not conducted at baseline. Hence, dmfs data from the 12-month visit served as the reference value. ^3^ Exponentiated estimate from generalized linear mixed model with negative binomial distribution is presented.

**Table 2:** Baseline Health Literacy (HL) as a Predictor of Change over Time in the Oral Health Constructs ^1^.

Oral Health Construct	Adjusted HL Z-Score Estimate (95% CI)	*p* Value	*p* Value for Adjusted HL Z-Score by Time Interaction
Parental Oral Health Knowledge ^2^	3.72 (2.67, 4.76)	<0.001	0.326
Parental Oral Health Beliefs Extended Health Belief Model			
Perceived Susceptibility	−0.19 (−0.28, −0.10)	<0.001	0.868
Perceived Severity	0.11 (0.05, 0.18)	<0.001	0.374
Perceived Barriers	−0.24 (−0.31, −0.16)	<0.001	0.519
Perceived Benefits	0.12 (0.06, 0.18)	<0.001	0.529
Self-efficacy	0.92 (0.61, 1.24)	<0.001	0.581
Locus of control (LOC)			
Internal LOC	0.09 (0.01, 0.16)	<0.001	0.770
External LOC—Powerful Others ^2^	−0.25 (−0.34, −0.15)	<0.001	0.407
External LOC—Chance	−0.29 (−0.38, −0.20)	<0.001	<0.001
Parental Oral Health Behavior ^3^	2.75 (0.97, 4.53)	<0.001	0.965
Pediatric Oral Health Outcomes			
Oral Health Status	−0.09 (−0.17, −0.002)	0.004	0.747
dmfs ^3^	1.15 (0.68, 1.95)	0.838	0.332

^1^ For each oral health construct, the table displays the estimate, 95% confidence interval (CI), and *p* value for the health literacy z-score as well as the overall *p* value for the health literacy z-score by time interaction across all time points. Detailed time point-specific comparisons are provided in Supplemental Table S2. Models were adjusted for parent’s age and income at baseline as well as child’s gender. ^2^ Because these constructs showed significant treatment group by time interactions, the final models for these constructs included treatment group and the interaction of treatment group by time as covariates. ^3^ Exponentiated estimate from generalized linear mixed model with negative binomial distribution is presented. Because behavior items collected at baseline differed from those collected at the follow-up time points, we excluded baseline behavioral data and used 12-month data as the reference value. Given that children were enrolled as newborns, dental evaluations were not conducted at baseline. Hence, dmfs data from the 12-month visit served as the reference value.

**Table 3:** Change over Time in External LOC—Chance by Health Literacy Category ^1^.

Oral Health Construct	Adjusted Time Estimate (∆) (95% CI)	*p* Value
Low Health Literacy Group		
12 Months	−0.03 (−0.13, 0.12)	0.967
24 Months	−0.26 (−0.39, −0.14)	<0.001
36 Months	−0.15 (−0.29, −0.02)	0.023
High Health Literacy Group		
12 Months	−0.01 (−0.14, 0.13)	0.907
24 Months	0.09 (−0.05, 0.24)	0.201
36 Months	0.02 (−0.12, 0.17)	0.769

^1^ The table displays estimates comparing each time point to the reference time point (i.e., baseline). Also displayed are 95% confidence intervals (CI), and *p* values for the comparison of each time point to the reference value. Models were adjusted for parental age and income at baseline as well as child’s gender.

## Data Availability

No new data were created or analyzed in this study. Data sharing is not applicable to this article.

## Supplementary Materials

The following tables are available online at https://www.mdpi.com/article/10.3390/ijerph18115633/s1, Table S1. Differences in Change over Time by Treatment Group. Table S2. Baseline Health Literacy (HL) as a Predictor of Change over Time in the Oral Health Constructs—Complete Results.
