# Supplementary material for: Influence of Parental Health Literacy on Change over Time in the Oral Health of American Indian Children
Source: Int J Environ Res Public Health. 2021 May 25;18(11):5633. doi: 10.3390/ijerph18115633 (PMC8197463; doi:10.3390/ijerph18115633)
Supplement: Supplementary file 1 [file ijerph-18-05633-s001.zip › ijerph-1179672-supplementary.pdf]

International Journal of
Environmental Research and Public Health

MDPI

Table S1. Differences in Change over Time by Treatment Group [1].

Oral Health Construct	Group * Time Interaction Estimate (95% CI)	P Value
**Parental Oral Health Knowledge**		**0.022**
Group * 12 Months	3.11 (0.93, 5.30)	0.005
Group * 24 Months	2.29 (-0.29, 4.87)	0.082
Group * 36 Months	0.77 (-1.66, 3.21)	0.532
**Parental Oral Health Beliefs**		
**Extended Health Belief Model**		
**Perceived Susceptibility**		**0.159**
Group * 12 Months	-0.13 (-0.33, 0.06)	0.184
Group * 24 Months	0.10 (-0.09, 0.30)	0.306
Group * 36 Months	-0.02 (-0.23, 0.20)	0.889
**Perceived Severity**		**0.334**
Group * 12 Months	0.07 (-0.07, 0.21)	0.347
Group * 24 Months	-0.01 (-0.16, 0.14)	0.890
Group * 36 Months	0.12 (-0.04, 0.28)	0.146
**Perceived Barriers**		**0.999**
Group * 12 Months	-0.01 (-0.17, 0.15)	0.900
Group * 24 Months	-0.01 (-0.17, 0.16)	0.934
Group * 36 Months	-0.01 (-0.17, 0.16)	0.925
**Perceived Benefits**		**0.770**
Group * 12 Months	0.02 (-0.14, 0.19)	0.775
Group * 24 Months	-0.04 (-0.20, 0.11)	0.585
Group * 36 Months	0.03 (-0.13, 0.20)	0.697
**Self-efficacy**		**0.951**
Group * 12 Months	0.16 (-0.50, 0.82)	0.631
Group * 24 Months	0.00 (-0.75, 0.76)	0.993
Group * 36 Months	0.09 (-0.66, 0.83)	0.821
**Locus of Control (LOC)**		
**Internal LOC**		**0.742**
Group * 12 Months	0.04 (-0.14, 0.22)	0.688
Group * 24 Months	0.06 (-0.12, 0.25)	0.509
Group * 36 Months	-0.03 (-0.22, 0.16)	0.761
**External LOC-Powerful Others**		**0.043**
Group * 12 Months	-0.22 (-0.41, -0.04)	0.017
Group * 24 Months	-0.21 (-0.40, -0.02)	0.032
Group * 36 Months	-0.09 (-0.29, 0.12)	0.401
**External LOC-Chance**		**0.436**
Group * 12 Months	-0.12 (-0.30, 0.06)	0.201
Group * 24 Months	-0.01 (-0.20, 0.18)	0.919
Group * 36 Months	-0.10 (-0.30, 0.09)	0.291
**Parental Oral Health Behavior** [2]		**0.927**
Group * 24 Months	-0.04 (-3.73, 3.65)	0.984
Group * 36 Months	0.60 (-3.18, 4.37)	0.757
**Pediatric Oral Health Outcomes**		
**Oral Health Status**		**0.468**
Group * 12 Months	-0.10 (-0.31, 0.11)	0.335
Group * 24 Months	-0.05 (-0.30, 0.21)	0.722

Oral Health Construct	Group * Time Interaction Estimate (95% CI)	P Value
Group * 36 Months	-0.17 (-0.44, 0.09)	0.194
**Dmfs** [2]		**0.934**
Group * 24 Months	1.12 (0.53, 2.37)	0.769
Group * 36 Months	1.03 (0.49, 2.18)	0.940

[1] For each construct, the table displays estimates of the treatment group by time interaction comparing each time point to the reference time point and reference group (control group). Unless otherwise specified, the reference time point is baseline. Also displayed are 95% confidence intervals (CI), p values for the comparison to the reference value, and overall p values for the time by group interaction. Models were adjusted for parental age and income at baseline as well as child's gender. [2] Exponentiated estimate from generalized linear mixed model with negative binomial distribution is presented. Because behavior items collected at baseline differed from those collected at the follow-up time points, we excluded baseline behavioral data and used 12-month data as the reference value. Given that children were enrolled as newborns, dental evaluations were not conducted at baseline. Hence, dmfs data from the 12-month visit served as the reference value.

Table S2. Baseline Health Literacy (HL) as a Predictor of Change over Time in the Oral Health Constructs – Complete Results [1].

Oral Health Construct	Adjusted HL Z-score Estimate (95% CI)	P value	Adjusted Time Estimate (Δ) (95% CI)	P value	Adjusted HL Z-score by Time Interaction Estimate (95% CI)	P value
**Parental Oral Health Knowledge** [2]	3.72 (2.67, 4.76)	**<0.001**		**<0.001**		**0.326**
12 Months			4.92 (3.37, 6.46)	<0.001	-0.84 (-1.97, 0.28)	0.142
24 Months			4.84 (3.00, 6.67)	<0.001	-1.18 (-2.54, 0.17)	0.087
36 Months			5.28 (3.54, 7.02)	<0.001	-0.64 (-1.93, 0.64)	0.325
**Parental Oral Health Beliefs**						
**Extended Health Belief Model**						
**Perceived Susceptibility**	-0.19 (-0.28, -0.10)	**<0.001**		**0.251**		**0.868**
12 Months			-0.09 (-0.19, 0.004)	0.061	-0.02 (-0.13, 0.08)	0.645
24 Months			-0.05 (-0.15, 0.05)	0.356	-0.03 (-0.13, 0.07)	0.593
36 Months			-0.02 (-0.12, 0.09)	0.781	0.01 (-0.10, 0.12)	0.870
**Perceived Severity**	0.11 (0.05, 0.18)	**<0.001**		**0.136**		**0.374**
12 Months			0.03 (-0.04, 0.10)	0.427	-0.04 (-0.11, 0.04)	0.309
24 Months			0.01 (-0.07, 0.08)	0.869	-0.02 (-0.11, 0.06)	0.542
36 Months			-0.06 (-0.14, 0.02)	0.144	0.03 (-0.06, 0.11)	0.528
**Perceived Barriers**	-0.24 (-0.31, -0.16)	**<0.001**		**0.193**		**0.519**
12 Months			0.03 (-0.05, 0.11)	0.414	0.05 (-0.03, 0.14)	0.213
24 Months			0.02 (-0.07, 0.10)	0.720	0.06 (-0.03, 0.15)	0.169
36 Months			0.08 (-0.002, 0.17)	0.056	0.05 (-0.04, 0.14)	0.245
**Perceived Benefits**	0.12 (0.06, 0.18)	**<0.001**		**0.343**		**0.529**
12 Months			0.05 (-0.03, 0.14)	0.232	0.00 (-0.09, 0.08)	0.927
24 Months			0.05 (-0.03, 0.13)	0.181	-0.05 (-0.13, 0.03)	0.251
36 Months			0.07 (-0.01, 0.16)	0.082	-0.05 (-0.13, 0.04)	0.282
**Self-efficacy**	0.92 (0.61, 1.24)	**<0.001**		**0.428**		**0.581**
12 Months			0.18 (-0.16, 0.51)	0.302	-0.21 (-0.56, 0.13)	0.225
24 Months			-0.09 (-0.47, 0.29)	0.632	-0.02 (-0.42, 0.37)	0.917
36 Months			-0.03 (-0.41, 0.35)	0.865	-0.06 (-0.45, 0.34)	0.779

Oral Health Construct	Adjusted HL Z-score Estimate (95% CI)	P value	Adjusted Time Estimate (Δ) (95% CI)	P value	Adjusted HL Z-score by Time Interaction Estimate (95% CI)	P value
**Locus of control (LOC)**						
**Internal LOC**	0.09 (0.01, 0.16)	**<0.001**		**0.013**		**0.770**
12 Months			0.14 (0.04, 0.23)	0.003	0.00 (-0.09, 0.09)	0.978
24 Months			0.03 (-0.06, 0.12)	0.525	0.04 (-0.06, 0.14)	0.404
36 Months			0.05 (-0.05, 0.14)	0.303	0.03 (-0.07, 0.13)	0.602
**External LOC-Powerful Others** [2]	-0.25 (-0.34, -0.15)	**<0.001**		**<0.001**		**0.407**
12 Months			-0.35 (-0.48, -0.22)	<0.001	0.01 (-0.08, 0.11)	0.762
24 Months			-0.46 (-0.59, -0.32)	<0.001	0.08 (-0.02, 0.18)	0.110
36 Months			-0.36 (-0.50, -0.21)	<0.001	0.04 (-0.07, 0.15)	0.441
**External LOC-Chance**	-0.29 (-0.38, -0.20)	**<0.001**		**0.062**		**<0.001**
12 Months			-0.01 (-0.10, 0.09)	0.891	0.02 (-0.08, 0.11)	0.736
24 Months			-0.11 (-0.21, -0.02)	0.019	0.22 (0.12, 0.32)	<0.001
36 Months			-0.08 (-0.18, 0.02)	0.105	0.14 (0.03, 0.24)	0.009
**Parental Oral Health Behavior** [3]	2.75 (0.97, 4.53)	**<0.001**		**<0.001**		**0.965**
24 Months			-7.64 (-9.54, -5.74)	<0.001	0.25 (-1.73, 2.23)	0.802
36 Months			-7.67 (-9.60, -5.73)	<0.001	0.07 (-1.97, 2.10)	0.950
**Pediatric Oral Health Outcomes**						
**Oral Health Status**	-0.09 (-0.17, -0.002)	**0.004**		**<0.001**		**0.747**
12 Months			0.30 (0.20, 0.41)	<0.001	-0.05 (-0.16, 0.06)	0.348
24 Months			0.92 (0.79, 1.04)	<0.001	-0.02 (-0.15, 0.12)	0.821
36 Months			1.13 (1.00, 1.27)	<0.001	0.00 (-0.14, 0.14)	0.970
**dmfs** [3]	1.15 (0.68, 1.95)	**0.838**		**<0.001**		**0.332**
24 Months			15.92 (10.73, 23.61)	<0.001	0.82 (0.54, 1.26)	0.361
36 Months			67.36 (45.06, 100.68)	<0.001	0.73 (0.48, 1.12)	0.147

[1] For each oral health construct, the table displays the estimate of the health literacy z-score by time interaction comparing each time point to the reference time point. Unless otherwise specified, the reference time point is baseline. Also displayed are 95% confidence intervals (CI), p values for the comparison of each time point to the reference value, and overall p values across all time points. Models were adjusted for parent's age and income at baseline as well as child's gender. [2] Because these constructs showed significant treatment group by time interactions, the final models for these constructs included treatment group and the interaction of treatment group by time as covariates. [3] Exponentiated estimate from generalized linear mixed model with negative binomial distribution is presented. Because behavior items collected at baseline differed from those collected at the follow-up time points, we excluded baseline behavioral data and used 12-month data as the reference value. Given that children were enrolled as newborns, dental evaluations were not conducted at baseline. Hence, dmfs data from the 12-month visit served as the reference value.
